# Human Bone Marrow Stromal Cells: A Reliable, Challenging Tool for *In Vitro* Osteogenesis and Bone Tissue Engineering Approaches

**DOI:** 10.1155/2016/7842191

**Published:** 2016-05-11

**Authors:** Ute Hempel, Katrin Müller, Carolin Preissler, Carolin Noack, Sabine Boxberger, Peter Dieter, Martin Bornhäuser, Manja Wobus

**Affiliations:** ^1^Institute of Physiological Chemistry, Medical Faculty Carl Gustav Carus, Technical University Dresden, 01307 Dresden, Germany; ^2^Stem Cell Lab, Medical Clinic I, University Hospital Carl Gustav Carus, Technical University Dresden, 01307 Dresden, Germany

## Abstract

Adult human bone marrow stromal cells (hBMSC) are important for many scientific purposes because of their multipotency, availability, and relatively easy handling. They are frequently used to study osteogenesis* in vitro*. Most commonly, hBMSC are isolated from bone marrow aspirates collected in clinical routine and cultured under the “aspect plastic adherence” without any further selection. Owing to the random donor population, they show a broad heterogeneity. Here, the osteogenic differentiation potential of 531 hBMSC was analyzed. The data were supplied to correlation analysis involving donor age, gender, and body mass index. hBMSC preparations were characterized as follows: (a) how many passages the osteogenic characteristics are stable in and (b) the influence of supplements and culture duration on osteogenic parameters (tissue nonspecific alkaline phosphatase (TNAP), octamer binding transcription factor 4, core-binding factor alpha-1, parathyroid hormone receptor, bone gla protein, and peroxisome proliferator-activated protein *γ*). The results show that no strong prediction could be made from donor data to the osteogenic differentiation potential; only the ratio of induced TNAP to endogenous TNAP could be a reliable criterion. The results give evidence that hBMSC cultures are stable until passage 7 without substantial loss of differentiation potential and that established differentiation protocols lead to osteoblast-like cells but not to fully authentic osteoblasts.

## 1. Introduction

Human bone marrow stromal cells (hBMSC; usual synonyms bone marrow stem cells, mesenchymal stromal cells, and mesenchymal stem cells) are widely used for tissue engineering approaches, to evaluate the performance of diverse biomaterials, to study tissue development and regeneration, and to understand molecular issues of cell differentiation [1, 2]. The cells are available in reasonable to large amounts, often from bone marrow donation surpluses, and they are relatively simple to handle* in vitro*. Pittenger et al. [3, 4] described the clonal multilineage potential of hBMSC and published first characterization criteria and differentiation protocols. Accordingly, hBMSC can be differentiated* in vitro* into cells with osteogenic, adipogenic, or chondrogenic features by adding particular supplements to the culture medium. The International Society for Cellular Therapy (ISCT) described established, clearly defined minimal characteristics of multipotent mesenchymal stromal cells: (1) adherence to cell culture plastic under standard conditions, (2) expression of certain surface markers (CD105, CD73, and CD90), (3) absence of CD45, CD34, CD11b or CD14, CD79*α* or CD19, and HLA-DR, and (4) trilineage differentiation capacity* in vitro* confirmed by staining of mineralized nodules (osteoblasts), collagen type II or glycosaminoglycans (chondrocytes), and lipid droplet accumulation (adipocytes) [5–7]. McNiece [8] demonstrated that plastic adherence is an adequate but not mandatory criterion for BMSC characterization. Beside the “minimal essential markers” propagated by ISCT, a broad panel of other markers as reviewed [9–12] was used for BMSC identification and characterization, supplemented recently by leptin receptor as a marker of osteogen/adipogen predetermined cells in the bone marrow niche [13]. hBMSC populations isolated from bone marrow aspirates revealed a broad heterogeneity predetermined by actual bone marrow niche composition, by the method of harvest, and by cell culture conditions and most of all by the individual donor variation [9, 14, 15]. Although most of the hBMSC preparations meet the “minimal essential markers” and are used without any further characterization or isolation of subpopulations, the individual differentiation capacity of each hBMSC preparation and the* in vitro* prospects (passaging, differentiation strategies) of the cells should to be taken into account. The donor variability cannot be influenced but should also be attended to.

The rationale for this study was to identify correlations between the differentiation behavior of hBMSC and the donor population which would allow a prediction of differentiation capacity of individual cell preparations. The study focuses on the osteogenic differentiation potential of hBMSC as one of the “minimal essential criterions” and one of the most clinically relevant features. For this purpose >500 hBMSC preparations were differentiated* in vitro* in the presence of osteogenic supplements according to a standardized protocol and examined in the second passage for their activity of tissue nonspecific alkaline phosphatase (TNAP). The quantity of the cohort enabled a solid and extensive statistical analysis of the data considering different aspects. TNAP values were subjected to correlation analyses to analyze relations to donor age, gender, and body mass index (BMI). For a more detailed characterization of the* in vitro* behavior, the influence of osteogenic supplements on particular osteogenic markers was investigated over 25 days with four defined hBMSC preparations. Furthermore, the influence of multiple passaging on these parameters was examined.

## 2. Materials and Methods

### 2.1. Isolation and Cultivation of Human Bone Marrow Stromal Cells (hBMSC)

Bone marrow aspirates were collected from Caucasian donors at the Dresden Bone Marrow Transplantation Centre of the University Hospital Carl Gustav Carus. The donors fulfilled the standards for bone marrow donation (e.g., free of HIV, HBV, and serious illness), were informed, and gave their approval. The study was approved by the local ethics commission (EK263122004, EK114042009). hBMSC were isolated according to Oswald et al. [16]. For preparation details see supplemental information in Supplementary Material available online at http://dx.doi.org/10.1155/2016/7842191. When the cells reached about 90% confluence, they were trypsinized with 0.05% trypsin/0.05% EDTA (v/v) (Gibco) in PBS. The passage 1 (P1) cells were transferred into freezing medium containing 90% of human serum albumin solution (dissolved to 50 g/L in PBS, Braun, Melsungen, Germany) and 10% of dimethylsulfoxide (Wak Chemie Medical GmbH, Steinbach, Germany) and stored in liquid nitrogen until subcultivation (the first subculture is denoted as P2). The origin cell populations were all positive for CD73, CD90, and CD105 and negative for CD14, CD43, CD45, and CD133 [16].

The hBMSC preparations in the study were taken as they accrued in the Bone Marrow Transplantation Center and not specifically selected. The bone marrow aspirates were always handled and prepared in standardized manner and the following cell culture and analyses were performed under standardized conditions.

*Osteogenic Differentiation Potential*. 10,000 hBMSC per well of a 24-well TCPS plate (5,555 hBMSC/cm^2^) were plated in 1 mL of DMEM containing 10% of HI-FCS (Biochrom, Berlin, Germany) (= basic medium, BM). At day 4 after plating the medium was changed by 1 mL of BM and in parallel by 1 mL BM supplemented with 10 mM *β*-glycerophosphate (Sigma, Taufkirchen, Germany), 300 *μ*M ascorbate (Sigma), and 10 nM dexamethasone (dex) (Sigma) (= osteogenic differentiation medium, OM/D). Medium change was performed twice per week. At indicated time points, the medium was aspirated; cells were washed three times with PBS and stored deep-frozen at −80°C until they were analyzed for activity of tissue nonspecific alkaline phosphatase (TNAP) or gene expression.

*Influence of Passaging on Cell Number and Doubling Time*. hBMSC of P1 were plated in T75 TCPS culture flasks (250,000 hBMSC/T75 flask) in 10 mL of BM (= P2). For determination of cell number, hBMSC were trypsinized after 7 days and counted in a Neubauer chamber. Then 250,000 hBMSC of P2 per T75 flask were reseeded in BM again for 7 days. This procedure was repeated until P9. The average doubling time for each passage was calculated from cell counted numbers accordingly by the equation (168 h*∗*log⁡2)/(log⁡*N* − log⁡*N*_0_) (*N*, number of cells after 7 days; *N*_0_, 250,000 cells) (see supplemental Figure 4S) [17].

*Influence of Passaging on Osteogenic Differentiation Potential*. hBMSC of P1 until P9 were plated in 24-well TCPS plates (10,000 hBMSC per well in 1 mL of BM for TNAP activity) or in 3 cm TCPS petri dishes (40,000 hBMSC per 3 cm petri dish in 2 mL cell of BM for gene expression analyses). hBMSC were cultured for 4 days in BM and then until day 15 in parallel in BM or in OM/D; medium was changed twice per week.

### 2.2. Activity of Tissue Nonspecific Alkaline Phosphatase (TNAP)

TNAP activity was determined from cell lysates (TNAP lysis buffer: 1.5 M Tris-HCl, pH 10 containing 1 mM ZnCl_2_, 1 mM MgCl_2_, and 1% Triton X-100) with p-nitrophenylphosphate as a substrate as described [18]. TNAP activity was calculated from a linear calibration curve (*r* > 0.99) prepared with p-nitrophenol. Protein concentration of the lysate was determined with RotiQuant protein assay (Roth GmbH, Karlsruhe, Germany) and was calculated from a linear calibration curve (*r* > 0.99) obtained with bovine serum albumin. Specific TNAP activity is given in mU/mg protein.

### 2.3. Gene Expression Analysis

For the analysis of gene expression, RNA was prepared using RNeasy Mini Kit (Qiagen, Hilden Germany). cDNA was synthesized using the QuantiTect Reverse Transcription Kit (Qiagen) including a DNA digestion step. Real time PCR reactions were performed using RotorGene PG-3000 PCR 854 machine (Corbett, Wasserburg, Germany) with the RotorGene SYBR Green PCR Kit (Qiagen). For detailed PCR conditions see Table 1 and supplemental information. The relative expression values were counted using the comparative quantification method of the RotorGene software release 6.0. For quantitation, values are normalized to hBMSC in P2 in BM (passaging) or hBMSC in BM at day 1 (time course).

### 2.4. Statistics

All statistical analyses were performed with GraphPad Prism 5.04 software (Statcon, Witzenhausen, Germany). Statistical significance was analyzed by *t*-test and one-way or two-way ANOVA with Bonferroni's post-test. Correlation coefficients were calculated by regression analysis. Donor frequency versus inducibility was analyzed by nonlinear curve fit (Gaussian distribution). More detailed information about the analyzed data and the applied tests is given in Results and the figure legends.

## 3. Results

### 3.1. Osteogenic Differentiation Potential of Human Bone Marrow Stromal Cells (hBMSC): Evaluation of Donor Variation

The aim of this experimental setting was to evaluate the osteogenic differentiation potential of hBMSC* in vitro* and to determine how reliably hBMSC differentiate into osteoblast-like cells. Furthermore it was determined if the osteogenic differentiation potential of the hBMSC preparations depends on individual factors such as donor age, gender, or BMI. The information about the donors (number, age, gender, and BMI) is given in Table 2. hBMSC from 531 donors (31.6% females, 68.4% males; mean age 32.0 ± 0.4 yrs.) in the second passage were analyzed. For a refined biometrical analysis, the entire cohort was divided into three groups (whole donor group, females, and males) and further subdivided by the criterion “donor age” considering that the groups consists of approximately the same number of donors. In these age subgroups (18–25 yrs., 26–35 yrs., and >35 yrs.) the proportion of females and males was not significantly different compared to the entire population. After culturing the hBMSC for 15 days in basic medium (BM) or with osteogenic supplements (*β*-glycerophosphate, ascorbate, and dex; OM/D) [4], TNAP activity was determined as a marker for osteogenic differentiation. Another frequently used characteristic of osteogenic differentiated hBMSC, the accumulation of calcium phosphate, was not determined in this study since it was shown earlier that TNAP activity correlates well with calcium phosphate deposition [19].

**Figure 1:**
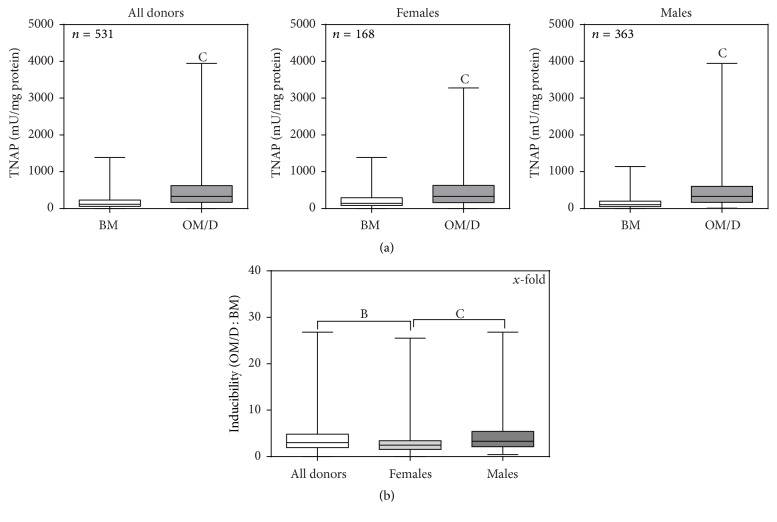
Osteogenic differentiation potential of hBMSC (TNAP activity and TNAP inducibility). hBMSC in passage 1 were plated in BM (basic medium) onto TCPS (= P2). From day 4 after plating, the cells were cultured either in BM or in OM/D (osteogenic differentiation medium) and analyzed at day 15 after plating for TNAP activity with p-nitrophenylphosphate as a substrate; the calculation of TNAP activity was performed with a linear calibration curve obtained with p-nitrophenolate (*r* = 0.9979). (a) TNAP activity was normalized to the protein content of the lysates and plotted for all donors and female and male donors, separately. Significant differences of OM/D mean versus BM mean values were analyzed by paired *t*-test and indicated with C (*p* < 0.001); *n* = 531 (all donors), *n* = 168 (females), and *n* = 363 (males). (b) The inducibility of TNAP activity (TNAP activity in OM/D : TNAP activity in BM) was calculated from individual donor data pairs and presented for all donors, females and males as well. Significant differences of mean values were analyzed by one-way ANOVA with Bonferroni's post-test and indicated with B (*p* < 0.01) and C (*p* < 0.001); *n* = 531 (all donors), *n* = 168 (females), and *n* = 363 (males).

Figure 1(a) shows the TNAP activity in BM and in OM/D for all donors, the female donors, and the male donors. Although the minimum/maximum TNAP values as well as the 25%/75% percentiles varied in a wide range, the induction of TNAP activity by OM/D was highly significant (*p* < 0.001) for all groups. Figure  1S1 (supplemental data) shows that also in all age subgroups the induced TNAP activity (OM/D) was significantly higher than the endogenous TNAP activity (BM). For an easier evaluation of the osteogenic differentiation potential, we defined the value “inducibility” which is calculated from the quotient of induced TNAP activity to endogenous TNAP activity. Accordingly, the results are presented in Figure 1(b) and Table 2 and demonstrate that the mean inducibility in the entire group was 3.97 ± 0.16. The female donors had a significant lower inducibility (3.01 ± 0.25) than the whole cohort and the male donors (4.41 ± 0.20). The data were further analyzed concerning differences of female donors to male donors with regard to TNAP activity in BM and OM/D and inducibility (Figure 1S2). It was seen that endogenous TNAP activity was significantly lower in the 26–35-year male donors compared to the corresponding female subgroup. The significant difference of TNAP inducibility between female donors and male donors (all donors) was also valid also for the 18–25-year and the 26–35-year subgroup but not in the >35-year subgroup. In a second approach, the age subgroups were compared with regard to TNAP activity in BM and OM/D and inducibility (Figure 1S3). In all age subgroups, no significant differences were observed for TNAP activity in BM and OM/D. The inducibility differed significantly between the 18–25-year and the >35-year groups for all donors and male donors indicating a decreasing inducibility with increasing age in these groups.

**Figure 2:**
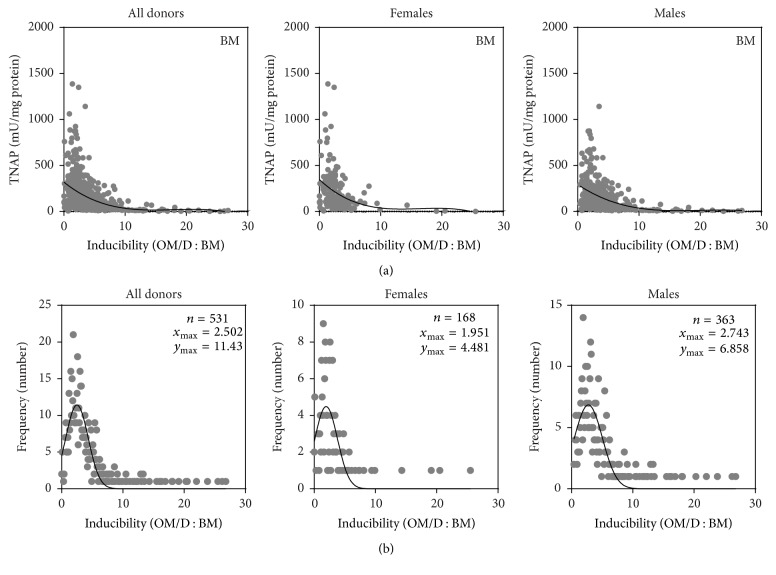
Correlation of TNAP inducibility to endogenous TNAP activity and frequency of certain inducibility values. hBMSC in passage 1 were plated in BM (basic medium) onto TCPS (= P2). From day 4 after plating, the cells were cultured either in BM or in OM/D (osteogenic differentiation medium) and analyzed at day 15 after plating for TNAP activity as described. The inducibility of TNAP activity (TNAP in OM/D : TNAP in BM) was calculated from individual donor data pairs and plotted against endogenous TNAP activity (a). The black line indicates a cubic curve fit; *R*-square values are given in Table 2; *n* = 531 (all donors), *n* = 168 (females), and *n* = 363 (males). By plotting the number of donors with a given TNAP inducibility, the frequency of certain inducibility value was figured out (b). The data points were analyzed by nonlinear curve fit and resulted in a Gaussian distribution. The peak characteristics (*x*_max_, *y*_max_) are indicated in the graphs; *n* = 531 (all donors), *n* = 168 (females), and *n* = 363 (males).

To see whether already endogenous TNAP values allow a prediction for inducibility and therefore an estimation of osteogenic differentiation potential of hBMSC, a correlation analysis was performed (Figure 2(a)). High endogenous TNAP activities correlated more frequently with low inducibility. The data were analyzed by a nonlinear regression analysis performing a cubic curve fit (see Table 2 for *R*-square values).

The minimum/maximum values of TNAP inducibility are distributed over a wide range: Some of the hBMSC preparations had an about 27-fold extreme high inducibility; on the other hand, some of the hBMSC preparations did not respond to osteogenic supplements; moreover they revealed slightly reduced TNAP values in OM/D compared to BM (Figures 1(b), 1S2, 1S3, and 2(a)).  Figure 2(b) shows the frequency of each inducibility value. These data fitted to a Gaussian distribution with maxima for inducibility at 2.5 (all donors), 1.95 (female donors), and 2.74 (male donors). Table 2 shows the inducibility range in which 68.3% of the donors fitted (calculated from mean ±  *δ* (= SD of Gaussian plot)) and the inducibility range in which 50% of the donors fitted (calculated from mean ± 0.675*δ*). Taking these data, it can be stated that the majority of the hBMSC preparations (≥50%) showed a moderate, 1.2-fold to 4.3-fold TNAP inducibility. hBMSC preparations above this range (>4.3-fold inducibility) are denoted as high responders. hBMSC preparations below 1.2-fold inducibility, concerned about 10% of all donors, are denoted as nonresponders or poor responders. By analyzing the age subgroups a tendency to lower inducibility values with increasing age was seen (see Table 2 and Figure 2S for details).

### 3.2. Correlation of Osteogenic Differentiation Potential (TNAP Activity, Inducibility) with Donor Data

**Figure 3:**
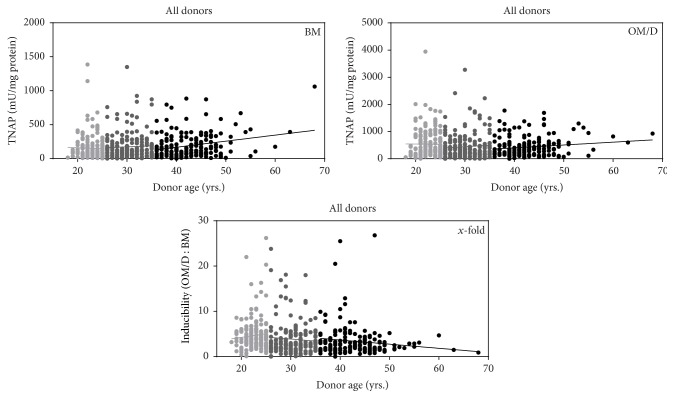
Correlation of donor age with TNAP activity and inducibility. hBMSC in passage 1 were plated in BM (basic medium) onto TCPS (= P2). From day 4 after plating, the cells were cultured either in BM or in OM/D (osteogenic differentiation medium). At day 15 after plating, the cells were analyzed for TNAP activity as described above. Data for endogenous TNAP activity, TNAP activity in OM/D, and TNAP inducibility for each donor age group were plotted each in one graph. The lines indicate linear curve fit. Regression characteristics (*p*-value, *R*-square) and the number of analyzed donors are given in Table 2.

To identify a correlation of TNAP activity and/or inducibility with donor data (which would allow approximatively a prediction of osteogenic differentiation potential) TNAP and inducibility data were plotted against donor age and body mass indices (BMI).  Figure 3 shows the correlation analysis of donor age with TNAP activity in BM and OM/D and inducibility. For each age subgroup a separate linear regression analysis was performed. Although some of the correlations were identified as significant (TNAP in BM: entire group and >35-year group; inducibility: entire group), the calculated correlation coefficients did not indicate a strong correlation (meaning *R*-square ≥0.7) with the donor age. The same results were found when male donors and female donors were analyzed; these data are presented in Table 2 and Figure 3S. In general, a tendency of an increased endogenous TNAP activity, a remaining TNAP activity in OM/D, and decreased inducibility with increasing donor age was noticed.

**Figure 4:**
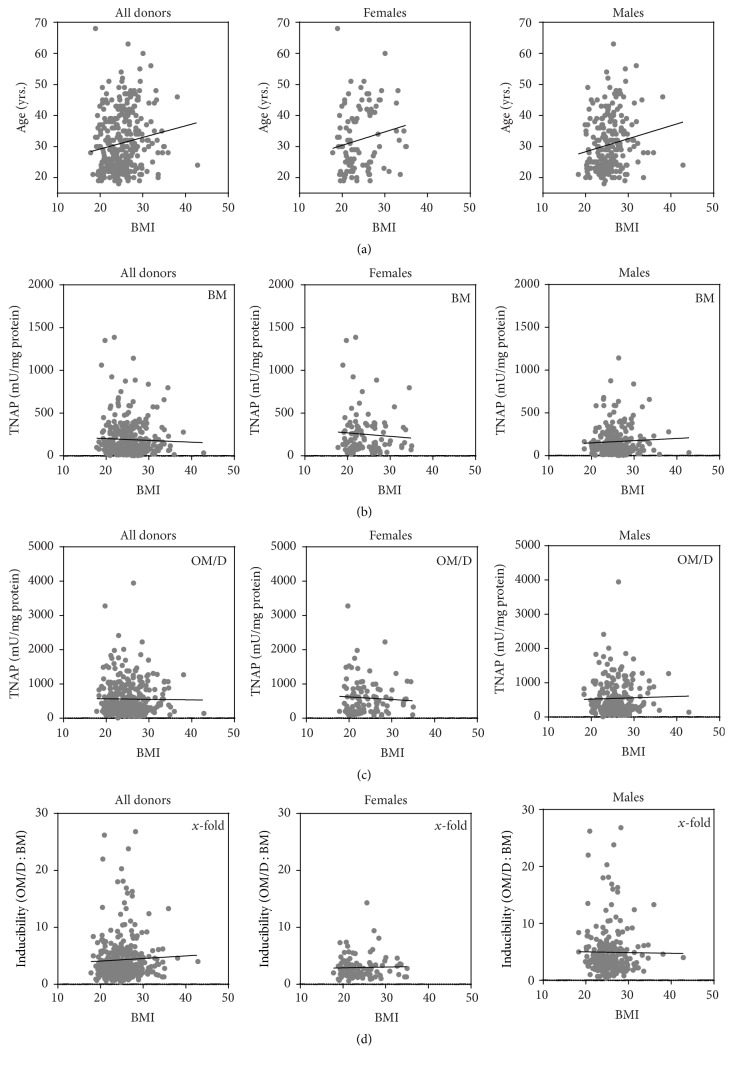
Correlation of body mass index with age, TNAP activity, and inducibility. hBMSC in passage 1 were plated in BM (basic medium) onto TCPS (= P2). From day 4 after plating, the cells were cultured either in BM or in OM/D (osteogenic differentiation medium). At day 15 after plating, the cells were analyzed for TNAP activity as described above. The data was presented for the entire group and females and males as well. Black lines indicate linear curve fit. Regression characteristics (*p*-value, *R*-square) and the number of analyzed donors are given in Table 2.

We have the information about body height and weight for about two-thirds of the donors (105 female donors, 233 male donors); therefore, TNAP data of 338 hBMSC were analyzed for a putative correlation with BMI. A positive correlation between donor age and BMI was determined in the male group and for all donors (Table 2, Figure 4(a)); however, this is without any relevance for osteogenic differentiation. No correlation was determined between TNAP activity in BM, OM/D or inducibility, and BMI (Table 2, Figure 4).

### 3.3. Influence of Culture Time on Particular Osteogenic Differentiation Parameters

**Figure 5:**
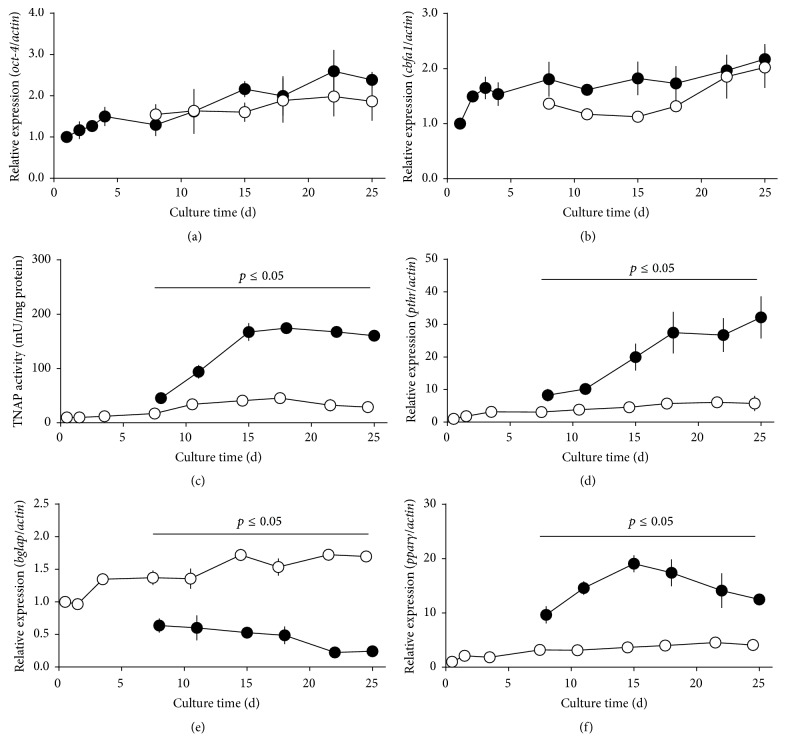
Influence of culture time and dexamethasone on hBMSC differentiation. hBMSC in passage 1 were plated in BM (basic medium) onto TCPS (= P2). From day 4 after plating, the cells were cultured either in BM (open circles) or in OM/D (osteogenic differentiation medium; black circles). At indicated time points (days 1, 2, and 4 in BM, and days 8, 11, 15, 18, 22, and 25 in both BM and OM/D) the cells were analyzed for the expression of octamer binding transcription factor 4 (*oct-4*, (a)), core-binding factor subunit alpha-1 (*cbfa1*, (b)), parathyroid hormone receptor (*pthr*, (d)), bone gla protein (*bglap*, (e)), and peroxisome proliferator-activated protein *γ* (*pparγ*, (f)). Relative gene expression (gene of interest) was assessed by real time PCR and normalized to the expression of beta-actin as house-keeping gene by comparative quantitation analysis setting day 1 in BM = 1. TNAP activity (c) was determined as described above. Significant differences of OM/D versus BM were analyzed for each time point with *t*-test and were indicated (*p* ≤ 0.05), *n* = 4.

hBMSC were cultured in P2 until day 25 in BM and from day 4 until day 25 in OM/D and analyzed at nine time points for TNAP activity and gene expression of octamer binding transcription factor 4 (*oct-4*), core-binding factor alpha-1 (*cbfa1*), parathyroid receptor (*pthr*), bone *γ*-carboxylglutamic acid-containing protein (*bglap*), and peroxisome proliferator-activated receptor *γ* (*pparγ*). For these experiments, four defined hBMSC preparations fitting in the 1.2–4.3-fold inducibility range were used. The transcription factor Oct-4 is frequently used as a marker of undifferentiated stem cells [20]; Cbfa1 is recognized as key transcription factor associated with osteoblast differentiation [21]. Both* oct-4* (Figure 5(a)) and* cbfa1* (Figure 5(b)) were expressed by hBMSC with a tendency to increase continuously (about 2-fold) over 25 days. The expression of* oct-4* and* cbfa1* was neither induced nor suppressed by OM/D. From previous work, it is known that dex is the most prominent agent to induce TNAP [19, 22]. TNAP activity of hBMSC increased significantly in OM/D and reached a maximal value at day 15 after plating (Figure 5(c)). Therefore, we analyzed the TNAP activity of all hBMSC preparations at day 15. Thereafter the TNAP activity remains almost constant with a slight tendency to decrease owing to the inhibitory effect of released phosphate [23]. A further characteristic of osteoblasts is the expression of parathyroid receptor [24, 25]. Binding of parathyroid hormone to its receptor activates the cyclic adenosine monophosphate (cAMP)/protein kinase A (PKA)/cAMP response element-binding protein-1 (CREB-1) signaling cascade and induces the expression of receptor activator of NF*κ*B ligand which facilitates osteoclastogenesis [26]. From day 8 until day 25,* pthr* expression in OM/D was continuously induced and significantly higher than in BM (Figure 5(d)). Bone *γ*-carboxylglutamic acid-containing protein (synonym osteocalcin) is responsible for controlling the bone mineralization process* in vivo* [27, 28] and has many other physiological functions, for example, on glucose regulation [10, 29]. The time course of* bglap* expression* in vitro* showed a gentle increase in BM over 25 days and a significant suppression in OM/D (Figure 5(e)). The expression of* pparγ*, an adipogenesis-controlling transcription factor which regulates fatty acid storage and glucose metabolism and which is partially involved in osteogenesis [10, 30–32], was induced in OM/D. From day 8 until day 25,* pparγ* expression was significantly higher in OM/D compared to BM and has a maximum at day 15 (Figure 5(f)).

### 3.4. Influence of Passaging on Cell Number, Doubling Time, and Osteogenic Differentiation Potential of hBMSC

The previous experimental setting evaluated the time course of particular differentiation parameters in dependence on medium supplements using cells in the same subculture (P2) over 25 days. In the following experiments the influence of multiple passaging (*in vitro* aging) on cell number, population doubling time, and osteogenic differentiation parameters was analyzed. The increase of cell numbers within seven days and resulting doubling time of hBMSC in dependence of passaging were examined from P2 until P10 (Figure 4S). Starting with 250,000 hBMSC in each passage, the cell number after seven days in BM remained relatively constant until P7 and decreased afterwards; the calculated doubling time behaved reversely.

**Figure 6:**
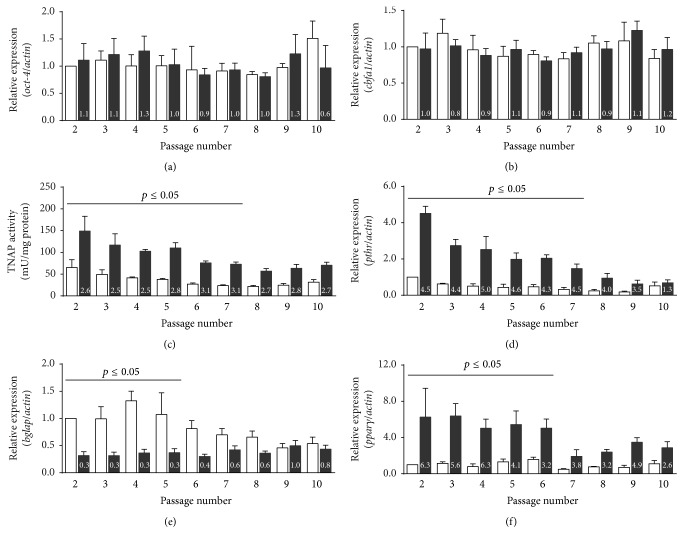
Influence of passaging on hBMSC differentiation. hBMSC in passage 1 until passage 9 were plated in BM (basic medium, white columns) onto TCPS (= P2–P10 for the analyses). From day 4 after plating, the cells were cultured either in BM or in OM/D (osteogenic differentiation medium, black columns). At day 15 after plating, the cells were analyzed for expression of* oct-4* (a),* cbfa1* (b),* pthr* (d),* bglap* (e), and* pparγ* (f), and for TNAP activity (c) as described above. Relative gene expression (gene of interest) was assessed by real time PCR and normalized to the expression of beta-actin as house-keeping gene by comparative quantitation analysis setting passage 2 value in BM = 1. Small digits in the black columns (OM/D) indicate the inducibility of the parameter in each passage as quotient OM/D : BM. Significant differences of OM/D versus BM for each passage were analyzed by two-way ANOVA with Bonferroni's post-test and indicated (*p* ≤ 0.05), *n* = 4.

The influence of passaging (P2 until P10) on osteogenic parameters was analyzed at day 15 in BM and OM/D. Continued passaging of hBMSC could result in (i) an alteration of absolute values of differentiation parameters and/or (ii) an altered inducibility of these parameters. Figures 6(a) and 6(b), respectively, show that the expression of* oct-4* and* cbfa1* maintained constant in BM and OM/D from P2 to P10; the ratio of OM/D to BM was around 1.0 (indicated with small digits in the OM/D columns) for both parameters. With increasing number of passages, the absolute values of TNAP activity decreased in BM and OM/D whereas the inducibility (quotient) remained constant (Figure 6(c)); until P7, the difference of induced TNAP to endogenous TNAP was significant. Similar to TNAP activity, the expression of* pthr* in OM/D was significantly higher until P7 than endogenous* pthr* expression. The inducibility of* pthr* expression decreased from 4.5 times at P2 to 3.5 times at P9 which is caused by a decreased* pthr* expression in OM/D and BM as well by increasing passaging (Figure 6(d)). At P10, the expression of* pthr* in BM increased resulting in an OM/D to BM ratio of 1.3. The OM/D to BM quotient for* bglap* expression was 0.3 and sustained until P5; >P5 the ratio increased caused by a decreasing* bglap* expression in BM (Figure 6(e)). The difference of* bglap* expression in OM/D to* bglap* expression in BM was significant from P2 until P5. The expression of pparg in OM/D exceeded its expression in BM significantly 6.3-fold in P2 to 3.2 fold in P6; >P6 the OM/D to BM quotient was maintained at 3.6 ± 0.5; however the absolute pparg expression values both in OM/D and in BM dropped down (Figure 6(f)).

## 4. Discussion

This study and data analyses were performed to examine aspects which could influence clinically relevant characteristics of hBMSC during* ex vivo* expansion, particularly osteogenic differentiation. The data were evaluated (i) with respect to donor variation, (ii) in order to identify a correlation of TNAP activity with donor age, gender, and BMI, (iii) to recognize the influence of osteogenic supplements on relevant osteoblast markers, (iv) to define the appropriate time point for analyzing relevant osteoblast markers, and (v) to get information on how many passages the hBMSC can be used in without substantial alteration of osteogenic differentiation potential.

The hBMSC isolated from a random donor cohort (one-third of female donors and two-thirds of male donors) met all minimal essential criteria given by ISCT [5]. The complete process from bone marrow harvest over hBMSC isolation until biochemical analyses was performed under standardized conditions by only few persons; nevertheless minimal variations during the harvesting procedure (puncture locus and depth) which could influence the further outcome of hBMSC cannot be completely excluded. Removing hBMSC from their original niche environment and culturing them on TCPS could induce spontaneous predifferentiation yielding in a heterogeneous of cell mixture [9, 33]. This finding together with the individual donor impact could be an explanation why hBMSC preparations revealed such a broad variability concerning the extent of their osteogenic differentiation. Phinney et al. [34] used in their study multiple bone marrow aspirates harvested from the same donor over half a year. Due to the immense differences found in the osteogenic differentiation potential of these cells even within one and the same person, the authors stated out that already the method of harvest can reasonably produce cell heterogeneity [34].

In the present study TNAP activity was used to evaluate the osteogenic differentiation capacity of hBMSC. This parameter represents an early differentiation marker of osteoblasts and is widely used because of its easy, high-throughput, low-budget, and reproducible determinability. TNAP releases phosphate from organic sources as prerequisite for proper mineralization which enables the osteoblast-facilitated formation of hydroxyapatite. It is known that an increase of TNAP activity induced by osteogenic supplements (ascorbate as an essential cofactor of prolyl-4-hydroxylase and lysyloxidase which are involved in the formation and maturation of collagens [35, 36], *β*-glycerophosphate as a substrate for TNAP* in vitro* [37], and dex as a regulator of* tnap* expression in hBMSC [38–41]) is a clear sign for hBMSC differentiating into the osteogenic lineage [4].

hBMSC in BM expressed endogenous TNAP activity in a range within 1–1386 mU/mg protein whereas in OM/D the TNAP activity varies within 2–2010 mU/mg protein. This broad range suggests that neither the endogenous nor the induced TNAP values alone can be used to estimate osteogenic differentiation potential of hBMSC* in vitro*. The goal of our study was to refine the criteria for choosing the “right cells” with a confidential osteogenic potential. Our data suggest that the quotient of induced to endogenous TNAP activity (*x*-fold, inducibility) could be a helpful additional criterion to predict the osteogenic potential of hBMSC. The majority of hBMSC exhibited an inducible TNAP activity in the range of 1.2 times to 4.3 times as determined from a Gaussian bell-curve fit. Applying the criterion “inducibility” would enable excluding non/poor- and hyperresponders from particular further studies. Thus, TNAP inducibility value could be a strong characteristic of osteogenic differentiation potential* a fortiori* when the absolute TNAP activity values either in BM or in OM/D vary in a broad range owing to the biological variation of the donors.

For a tentative prediction of osteogenic differentiation potential, TNAP activity and inducibility were correlated to each other and to donor age and BMI values. In tendency, a weak negative correlation of endogenous TNAP activity to inducibility was found suggesting that hBMSC with a very high endogenous TNAP activity should be considered critically since they are probably linked to the nonresponder/poor-responder group. The BMI of the donors had no influence on the osteogenic differentiation potential of the hBMSC. A positive correlation was seen between donor age and BMI in the male and in the entire group. The endogenous TNAP activity and the inducibility were in tendency slightly age-dependent: with increasing age the endogenous TNAP activity increased and the inducibility decreased. The results of the correlation studies suggest that although in some cases a significant dependence of the data exists the obtained correlation coefficients are quite too low as to be suitable enough for a strong prediction of osteogenic differentiation potential of hBMSC. The relationship of hBMSC characteristics (phenotype, proliferation, and differentiation) to donor age was investigated in several studies. As reviewed by Baker et al. [15], such studies were mostly performed with less than 50 donors. The study of Phinney et al. [34] performed with hBMSC from 17 donors showed that no correlation exists between proliferation rate, TNAP, and donor age or gender and that TNAP activity at day 8 and day 22 varied by a factor of 40 between the donors. Stolzing et al. [42] included 33 hBMSC preparations in their analysis and correlated the donor age (5–55 yrs.) to proliferation rate, TNAP level, and divers biochemical markers such as vitamin D receptor, glucocorticoid receptor, and apoptosis induction. The results indicated few significant differences between the very young donors (5–18 yrs.) and the adult donors (>18) including cell size (increasing with age), TNAP (decreasing with age), vitamin D receptor (decreasing with age), and glucocorticoid receptor (increasing with age). Siegel et al. [43] analyzed hBMSC of 52 donors between 13 and 80 years. They found a negative correlation for phenotype markers as CD 71, CD164, and CD 274 to donor age; for typical lineage commitment markers such as LPL, PPAR*γ*, OPN, TNAP, SOX-9, and collagen type II, however, neither a correlation to age nor significant differences between female and male donors were seen. Kim et al. [44] generated 32 hBMSC clones from a single donor and demonstrated that TNAP activity varies in each clone, altogether TNAP values covered about seven absorbency units. This result validates once again that beside the broad inter-bone marrow variance also a broad intra-bone marrow variance exists.

To characterize the influence of osteogenic supplements and to identify the correct time window for studying particular parameters, hBMSC with a comparable TNAP inducibility in the 1.2–4.3-fold range in P2 were cultured in parallel in BM and OM/D over 25 days and analyzed for TNAP activity and gene expression of osteoblast-associated markers. The examined parameters should characterize the hBMSC* in vitro* as stem cell-originated (*oct-4*) and osteogenic imprinted (*cbfa1*), and as osteoblast-like cells exhibiting early (TNAP activity) and late (*pthr, bglap*) osteoblast markers and as non-adipocyte-like cell (*pparg*) [11, 45]. The results show that hBMSC expressed* oct-4* and* cbfa1* and that these factors were not altered by dex. It was seen that TNAP activity and* pthr* expression were induced by dex. Furthermore, the expression of the osteogenic marker* bglap* was suppressed and the adipogenic marker* pparγ* was induced by dex suggesting that the established* in vitro* differentiation protocol results in non-absolutely authentic osteoblasts but in cells with many characteristic osteoblast features which should be denoted more correctly as osteoblast-like cells. Our results conformed to several other studies [10, 22, 31, 46]. Dex is a useful agent to generate osteoblast-like cells* in vitro*;* in vivo* it is rather known to impair bone formation and to cause osteoporosis, for example, by reducing collagen expression and decreasing vitamin D synthesis. Primarily glucocorticoids antagonize* in vivo* the effect of insulin on glucose and energy metabolism. An increasing number of studies discussed a positive effect of circulating Bglap on insulin secretion and insulin sensitivity (regulating glucose uptake, storage, and consumption); therefore in case of a glucose supply (*de novo*-synthesis, release) a downregulation of Bglap by dex seems to be a reasonable response [28, 29]. Dex has also a substantial impact on fatty acid utilization as seen by an increase in lipoprotein lipase, fatty acid binding protein, and* pparγ* expression in hBMSC [31] showing the ambivalence of this “osteogenic” supplement.

It is important for* in vitro* approaches to pay attention to the number of subcultivations the hBMSC can undergo without significantly changing their features. As reviewed by Kim et al. [44] the “*in vitro* age” (number of passages in culture) had a more pronounced effect on hBMSC fate* in vitro* than the “*in vivo* age” (physical age of the donors). Fickert et al. [47] analyzed the osteogenic differentiation potential of aged patients and stated no difference between hBMSC from old (>65 yrs.) compared to a younger (<50 yrs.) donors. An influence of* in vitro* aging on hBMSC behavior was described by Bonab et al. [48] who found that, beside a decrease of population doubling number and mean telomere length beginning in the ninth passage, a drop of differentiation potential became noticeable from the sixth passage. The hBMSC used in our study are primary, not immortalized cells which could be cultured and passaged not unlimited without loss of characteristic features and differentiation potential. To identify how many subcultures are feasible without substantial loss of* in vitro* behavior compared to P2, hBMSC with a comparable inducibility of TNAP activity were analyzed at day 15 for differentiation parameters. In summary, three effects with increasing passage number were seen: (i) no influence, either for absolute values or for inducibility (*cbfa1*, and* oct-4* expression); (ii) the decrease of absolute values in OM/D and BM with maintained inducibility (TNAP activity, and* pthr* expression excepted P10), and (iii) the decrease of absolute values in OM/D and or BM as well as alteration of inducibility (*bglap* and* pparγ* expression). Summarized, until P5 (latest until P7), hBMSC can be subcultured without any significant decline of osteogenic differentiation potential. Over P7 the cells tended to dedifferentiate and should not be used anymore for* in vitro* studies. These results are in line with the study of Bonab et al. [48] who used mineralization capacity to evaluate the influence of passaging on osteogenic differentiation potential of hBMSC. From investigations with MC3T3-E1 cells, it is known that this premature osteoblasts-reflecting cell line [49] also tends to dedifferentiate* in vitro*, however with the difference that the cells got exhausted not before passage 34 [50].

The critical discussion about* in vitro* and moreover* in vivo* potential of hBMSC is not finished yet. For putative clinical use there is a need to expand the cells* in vitro*. Beside the discussed problems, limitations and deficiencies in quality and also aging of the cells and spontaneous tumorigenesis are addressed [2, 51, 52]. The most frequently used, easiest, and fastest technology to get sufficient hBMSC for* in vitro* experiments is their isolation from fresh whole bone marrow without any further selection and subsequent expansion of plastic-adherent cells. The presented study suggests that, at least for common* in vitro* experiments, for example, evaluating hBMSC/osteoblast response to diverse substrates, biomaterials, or drugs, hBMSC can be used but donor variance,* in vitro* aging, and effects of culture medium supplements should be taken into account. Although the donor variance is a given fact and no strong prediction of differentiation potential from donor data seems to be possible, the conclusions from this study are as follows: (i) TNAP inducibility is a good selection criterion evaluating the osteogenic differentiation potential; about 70% of the examined hBMSC preparations had a TNAP inducibility in the 1.2–4.3-fold range; (ii) hBMSC preparations need a predifferentiation experiment to identify non-/poor and extreme responding cells; (iii) hBMSC preparations can be used until the fifth to seventh passage without any substantial loss of typical osteogenic characteristics; (iv) time course studies are useful to define correct time windows for particular parameters; and (v) in vitro osteogenic differentiation by established protocols leads to osteoblast-like cells revealing many features of authentic osteoblasts however lacking some others and expressing some adipogenic markers.

## 5. Conclusion

hBMSC are an easy-to-isolate and easy-to-handle but challenging source of primary osteoblast-like cells. Working with them, one should consider always not only the huge donor variance but also the fact of dedifferentiation* in vitro* (*in vitro* aging), the influence of medium supplements, and the appropriate time point for analyzing the parameter of interest. A prediction of osteogenic differentiation capacity from donor data (gender, age, and BMI) seems not to be possible.

## Supplementary Material

Materials and Methods: Isolation and cultivation of human bone marrow stromal cells (hBMSC) Gene expression analysis.
Figure S1. Osteogenic differentiation potential of hBMSC (TNAP activity) itemized to gender and age groups.
Figure S2. Correlation of TNAP inducibility to frequency of certain inducibility values itemized to age groups
Figure S3. Correlation of donor age with TNAP activity and inducibility itemized to gender.
Figure S4. Influence of passaging on cell number and doubling time.

## Figures and Tables

**Table 1:** Primer pairs used for real time PCR.

Gene^#^	Accession number	Forward primer	Reverse primer	Binding position	Product length (bp)	Product identity (%)^##^
*actin*	NM001101	aatgtggccgaggactttgattgc	ttaggatggcaagggacttcctgt	1414–1508	95	100
*oct-4*	NM203289	cttcaggagatatgcaaa	ccggttacagaacca	1367–1561	195	93
*cbfa1*	NM00102463	aatgacaccaccaggccaat	tggcctacaaaggtgggttt	3646–3805	160	100
*pthr*	NM000316	ttggcgtccactacattgtc	tccctggaaggagttgaaga	1453–1559	107	98
*bglap*	NM199173	ggtggagcctttgtgtccaagc	gtcagccaactcgtcacagtcc	169–327	159	97
*pparγ*	NM138711	tccgtggatctctccgtaat	ctgcaaccactggatctgtt	302–437	136	96

^#^Genes: *actin*,  *β*-actin; *oct-4*, octamer binding transcription factor 4; *cbfa*, core-binding factor subunit alpha-1; *pthr*, parathyroid hormone receptor; *bglap*, bone gla protein = osteocalcin; *pparγ*, *peroxisome proliferator-activated receptor gamma*.

^##^Determined by sequencing of amplified DNA products.

**Table 2:** Donor data (number, age, BMI, and TNAP activity) and correlation data.

Age groups/yrs.	All donors					Females					Males			
	**18–68**	18–25	26–35	>35		**18–68**	18–25	26–35	>35		**18–68**	18–25	26–35	>35
*Number of donors*														
Entire cohort	**531**	169	187	175		**168**	49	59	60		**363**	120	128	115

*Age of donors*														
Mean ± SEM/yrs.	**32.0 ± 0.39**	22.6 ± 0.14	30.2 ± 0.20	43.1 ± 0.39		**32.5 ± 0.74**	22.4 ± 0.27	29.6 ± 0.35	43.5 ± 0.75		**31.8 ± 0.46**	22.7 ± 0.16	30.5 ± 0.24	42.8 ± 0.45

*TNAP* ^a^ * activity in BM* ^b^														
Mean ± SEM/mU/mg protein	**176 ± 8.2**	156 ± 13.8	175 ± 14.4	196 ± 14.1		**215 ± 17.4**	196 ± 31.9	228 ± 31.7	217 ± 27.1		**158 ± 8.7**	139 ± 14.4	150 ± 14.8	185 ± 16.2
*TNAP activity in OM/D* ^c^														
Mean ± SEM/mU/mg protein	**470 ± 19.6**	535 ± 39.4	446 ± 34.5	434 ± 27.1		**460 ± 35.2**	532 ± 67.0	482 ± 74.4	380 ± 36.1		**475 ± 23.7**	536 ± 48.4	429 ± 37.0	462 ± 36.5

*TNAP inducibility* ^d^														
Mean ± SEM	**3.97 ± 0.16**	4.68 ± 0.28	3.87 ± 0.27	3.39 ± 0.27		**3.01 ± 0.25**	3.28 ± 0.31	2.71 ± 0.37	3.09 ± 0.53		**4.41 ± 0.20**	5.25 ± 0.37	4.40 ± 0.35	3.55 ± 0.30

*TNAP activity in BM versus inducibility*														
*R*-square	**0.1456**					**0.0997**					**0.1584**			

*Frequency versus inducibility (Gaussian fit)*														
*R*-square	**0.7055**	0.337	0.4116	0.3484		**0.3629**					**0.5709**			
Mean	**2.5**	3.46	2.15	2.02		**1.951**					**2.74**			
SD (*δ*)	**1.83**	2.54	2.15	1.87		**1.86**					**2.29**			
68.3% range	**0.67–4.34**	0.91–6.0	0–4.3	0.14–3.89		**0.1–3.81**					**0.45–5.03**			
50.0% range	**1.26–3.74**	1.74–5.17	0.69–3.6	0.75–3.28		**0.7–3.20**					**1.28–4.29**			

*TNAP in BM versus age*														
*p* value	0.0031^**∗**^	0.7729	0.5611	0.0009^**∗**^		**0.3741**	0.2692	0.5058	0.0726		0.0018^**∗**^	0.3910	0.4781	0.0063^**∗**^
*R*-square	**0.0164**	0.0005	0.0018	0.0618		**0.0048**	0.0259	0.0078	0.0545		**0.0266**	0.0662	0.0040	0.0641
*TNAP in OM/D versus age*														
*p* value	**0.0450**	0.2871	0.9025	0.3541		**0.2579**	0.5023	0.1167	0.2736		**0.6400**	0.7921	0.9956	0.0638
*R*-square	**0.0230**	0.0021	0.0001	0.0046		**0.0077**	0.0096	0.0426	0.0206		**0.0006**	0.0006	0.0001	0.0301

*Age versus inducibility*														
*p* value	0.0005^**∗**^	0.3024	0.3811	0.0748		**0.5715**	0.1116	0.6463	0.2421		0.0002^**∗**^	0.7497	0.2091	0.2224
*R*-square	**0.0227**	0.0064	0.0041	0.0182		**0.0020**	0.0530	0.0037	0.0024		**0.0368**	0.0009	0.0125	0.0132

*Number of donors*														
With BMI^e^ data	**338**					**105**					**233**			

*BMI*														
Mean ± SEM	**25.2 ± 0.20**					**24.1 ± 0.40**					**25.6 ± 0.23**			

*Age versus BMI*														
*p* value	0.0064^**∗**^					**0.0802**					0.0131^**∗**^			
*R*-square	**0.0219**					**0.0294**					**0.0264**			

*TNAP in BM versus BMI*														
*p* value	**0.4832**					**0.5138**					**0.4266**			
*R*-square	**0.0014**					**0.0042**					**0.0027**			
*TNAP in OM/D versus BMI*														
*p* value	**0.8312**					**0.5478**					**0.6799**			
*R*-square	**0.0001**					**0.0035**					**0.0007**			

*Inducibility versus BMI*														
*p* value	**0.4232**					**0.7769**					**0.8808**			
*R*-square	**0.0019**					**0.0008**					**0.0001**			

^a^TNAP, tissue nonspecific alkaline phosphatase; ^b^BM, basic medium; ^c^OM/D, osteogenic differentiation medium; ^d^inducibility calculated OMD : BM values from each donor separately; ^e^BMI, body mass index [weight [kg]/height [m]^2^].

^*∗*^Significant correlation; for further statistical analyses see figures and text.

## Abbreviations

Bglap: Bone *γ*-carboxylglutamic acid-containing protein
BM: Basic medium
BMI: Body mass index
cAMP: Cyclic adenosine monophosphate
Cbfa1: Core-binding factor subunit alpha-1
CREB-1: cAMP response element-binding protein-1
Dex: Dexamethasone
DMEM: Dulbecco's modified essential medium
DMSO: Dimethylsulfoxide
HBV: Hepatitis B virus
HIV: Human immunodeficiency virus
ISCT: International Society for Cellular Therapy
Oct-4: Octamer binding transcription factor 4
OM/D: Osteogenic differentiation medium
PBS: Phosphate-buffered saline
PCR: Polymerase chain reaction
PKA: Protein kinase A
PPAR*γ*: Peroxisome proliferator-activated receptor *γ*
PTHR: Parathyroid hormone receptor
SEM: Standard error of the mean
TCPS: Tissue culture polystyrene
TNAP: Tissue nonspecific alkaline phosphatase.

## References

[1] Ullah I., Subbarao R. B., Rho G. J. (2015). Human mesenchymal stem cells—current trends and future prospective. *Bioscience Reports*.

[2] D'souza N., Rossignoli F., Golinelli G. (2015). Mesenchymal stem/stromal cells as a delivery platform in cell and gene therapies. *BMC Medicine*.

[3] Pittenger M. F., Mackay A. M., Beck S. C. (1999). Multilineage potential of adult human mesenchymal stem cells. *Science*.

[4] Pittenger M. F. (2008). Mesenchymal stem cells from adult bone marrow. *Methods in Molecular Biology*.

[5] Dominici M., Le Blanc K., Mueller I. (2006). Minimal criteria for defining multipotent mesenchymal stromal cells. The International Society for Cellular Therapy position statement. *Cytotherapy*.

[6] Sousa B. R., Parreira R. C., Fonseca E. A. (2014). Human adult stem cells from diverse origins: an overview from multiparametric immunophenotyping to clinical applications. *Cytometry A*.

[7] Hayrapetyan A., Jansen J. A., van den Beucken J. J. J. P. (2015). Signaling pathways involved in osteogenesis and their application for bone regenerative medicine. *Tissue Engineering Part B Reviews*.

[8] McNiece I. (2007). Subsets of mesenchymal stromal cells. *Cytotherapy*.

[9] Pevsner-Fischer M., Levin S., Zipori D. (2011). The origins of mesenchymal stromal cell heterogeneity. *Stem Cell Reviews and Reports*.

[10] Zhang Y., Khan D., Delling J., Tobiasch E. (2012). Mechanisms underlying the osteo- and adipo-differentiation of human mesenchymal stem cells. *The Scientific World Journal*.

[11] Lv F.-J., Tuan R. S., Cheung K. M. C., Leung V. Y. L. (2014). Concise review: the surface markers and identity of human mesenchymal stem cells. *STEM CELLS*.

[12] Galderisi U., Giordano A. (2014). The gap between the physiological and therapeutic roles of mesenchymal stem cells. *Medicinal Research Reviews*.

[13] Zhou B. O., Yue R., Murphy M. M., Peyer J. G., Morrison S. J. (2014). Leptin-receptor-expressing mesenchymal stromal cells represent the main source of bone formed by adult bone marrow. *Cell Stem Cell*.

[14] Wagner W., Ho A. D. (2007). Mesenchymal stem cell preparations—comparing apples and oranges. *Stem Cell Reviews*.

[15] Baker N., Boyette L. B., Tuan R. S. (2015). Characterization of bone marrow-derived mesenchymal stem cells in aging. *Bone*.

[16] Oswald J., Boxberger S., Jørgensen B. (2004). Mesenchymal stem cells can be differentiated into endothelial cells in vitro. *Stem Cells*.

[17] Paul J. (1970). *Zell- und Gewebekulturen*.

[18] Hempel U., Hefti T., Kalbacova M., Wolf-Brandstetter C., Dieter P., Schlottig F. (2010). Response of osteoblast-like SAOS-2 cells to zirconia ceramics with different surface topographies. *Clinical Oral Implants Research*.

[19] Hempel U., Matthäus C., Preissler C., Möller S., Hintze V., Dieter P. (2014). Artificial matrices with high-sulfated glycosaminoglycans and collagen are anti-inflammatory and pro-osteogenic for human mesenchymal stromal cells. *Journal of Cellular Biochemistry*.

[20] Niwa H., Miyazaki J.-I., Smith A. G. (2000). Quantitative expression of Oct-3/4 defines differentiation, dedifferentiation or self-renewal of ES cells. *Nature Genetics*.

[21] Lian J. B., Javed A., Zaidi S. K. (2004). Regulatory controls for osteoblast growth and differentiation: role of Runx/Cbfa/AML factors. *Critical Reviews in Eukaryotic Gene Expression*.

[22] Hempel U., Möller S., Noack C. (2012). Sulfated hyaluronan/collagen I matrices enhance the osteogenic differentiation of human mesenchymal stromal cells in vitro even in the absence of dexamethasone. *Acta Biomaterialia*.

[23] Fernley H. N., Walker P. G. (1967). Studies on alkaline phosphatase: inhibition by phosphate derivatives and the substrate specificity. *Biochemical Journal*.

[24] Ben Azouna N., Jenhani F., Regaya Z. (2012). Phenotypical and functional characteristics of mesenchymal stem cells from bone marrow: comparison of culture using different media supplemented with human platelet lysate or fetal bovine serum. *Stem Cell Research & Therapy*.

[25] Jarocha D., Lukasiewicz E., Majka M. (2008). Adventage of Mesenchymal Stem Cells (MSC) expansion directly from purified bone marrow CD105+ and CD271+ cells. *Folia Histochemica et Cytobiologica*.

[26] Hofbauer L. C., Kühne C. A., Viereck V. (2004). The OPG/RANKL/RANK system in metabolic bone diseases. *Journal of Musculoskeletal Neuronal Interactions*.

[27] Bonucci E. (2012). Bone mineralization. *Frontiers in Bioscience*.

[28] Neve A., Corrado A., Cantatore F. P. (2013). Osteocalcin: skeletal and extra-skeletal effects. *Journal of Cellular Physiology*.

[29] Lombardi G., Perego S., Luzi L., Banfi G. (2015). A four-season molecule: osteocalcin. Updates in its physiological roles. *Endocrine*.

[30] Kawai M. (2013). Adipose tissue and bone: role of PPAR*γ* in adipogenesis and osteogenesis. *Hormone Molecular Biology and Clinical Investigation*.

[31] Noack C., Hempel U., Preissler C., Dieter P. (2015). Prostaglandin E_2_ impairs osteogenic and facilitates adipogenic differentiation of human bone marrow stromal cells. *Prostaglandins, Leukotrienes & Essential Fatty Acids*.

[32] Cao J., Ou G., Yang N. (2015). Impact of targeted PPAR*γ* disruption on bone remodeling. *Molecular and Cellular Endocrinology*.

[33] Wagner W., Ho A. D., Zenke M. (2010). Different facets of aging in human mesenchymal stem cells. *Tissue Engineering B*.

[34] Phinney D. G., Kopen G., Righter W., Webster S., Tremain N., Prockop D. J. (1999). Donor variation in the growth properties and osteogenic potential of human marrow stromal cells. *Journal of Cellular Biochemistry*.

[35] Padh H. (1990). Cellular functions of ascorbic acid. *Biochemistry and Cell Biology*.

[36] Peterkofsky B. (1991). Ascorbate requirement for hydroxylation and secretion of procollagen: relationship to inhibition of collagen synthesis in scurvy. *The American Journal of Clinical Nutrition*.

[37] Langenbach F., Handschel J. (2013). Effects of dexamethasone, ascorbic acid and *β*-glycerophosphate on the osteogenic differentiation of stem cells in vitro. *Stem Cell Research and Therapy*.

[38] Belvisi M. G., Wicks S. L., Battram C. H. (2001). Therapeutic benefit of a dissociated glucocorticoid and the relevance of in vitro separation of transrepression from transactivation activity. *The Journal of Immunology*.

[39] Ratman D., Vanden Berghe W., Dejager L. (2013). How glucocorticoid receptors modulate the activity of other transcription factors: a scope beyond tethering. *Molecular and Cellular Endocrinology*.

[40] Dezitter X., Fagart J., Taront S. (2014). A structural explanation of the effects of dissociated glucocorticoids on glucocorticoid receptor transactivation. *Molecular Pharmacology*.

[41] Hempel U., Preissler C., Vogel S. (2014). Artificial extracellular matrices with oversulfated glycosaminoglycan derivatives promote the differentiation of osteoblast-precursor cells and premature osteoblasts. *BioMed Research International*.

[42] Stolzing A., Jones E., McGonagle D., Scutt A. (2008). Age-related changes in human bone marrow-derived mesenchymal stem cells: consequences for cell therapies. *Mechanisms of Ageing and Development*.

[43] Siegel G., Kluba T., Hermanutz-Klein U., Bieback K., Northoff H., Schäfer R. (2013). Phenotype, donor age and gender affect function of human bone marrow-derived mesenchymal stromal cells. *BMC Medicine*.

[44] Kim Y. H., Yoon D. S., Kim H. O., Lee J. W. (2012). Characterization of different subpopulations from bone marrow-derived mesenchymal stromal cells by alkaline phosphatase expression. *Stem Cells and Development*.

[45] Bianco P. (2014). “Mesenchymal” stem cells. *Annual Review of Cell and Developmental Biology*.

[46] Li X., Jin L., Cui Q., Wang G.-J., Balian G. (2005). Steroid effects on osteogenesis through mesenchymal cell gene expression. *Osteoporosis International*.

[47] Fickert S., Schröter-Bobsin U., Gross A. F. (2011). Human mesenchymal stem cell proliferation and osteogenic differentiation during long-term ex vivo cultivation is not age dependent. *Journal of Bone and Mineral Metabolism*.

[48] Bonab M. M., Alimoghaddam K., Talebian F., Ghaffari S. H., Ghavamzadeh A., Nikbin B. (2006). Aging of mesenchymal stem cell in vitro. *BMC Cell Biology*.

[49] Kodama H. A., Amagai Y., Sudo H., Kasai S., Yamamoto S. (1981). Establishment of a clonal osteogenic cell line from newborn mouse calvaria. *Japanese Journal of Oral Biology*.

[50] Yan X.-Z., Yang W. Y., Yang F., Kersten-Niessen M., Jansen J. A., Both S. K. (2014). Effects of continuous passaging on mineralization of MC3T3-E1 cells with improved osteogenic culture protocol. *Tissue Engineering Part C: Methods*.

[51] Prockop D. J. (2009). Repair of tissues by adult stem/progenitor cells (MSCs): controversies, myths, and changing paradigms. *Molecular Therapy*.

[52] Savkovic V., Li H., Seon J.-K., Hacker M., Franz S., Simon J.-C. (2014). Mesenchymal stem cells in cartilage regeneration. *Current Stem Cell Research and Therapy*.

