# Supplementary material for: Human Bone Marrow Stromal Cells: A Reliable, Challenging Tool for In Vitro Osteogenesis and Bone Tissue Engineering Approaches
Source: Stem Cells Int. 2016 May 11;2016:7842191. doi: 10.1155/2016/7842191 (PMC4879246; doi:10.1155/2016/7842191)
Supplement: Supplementary file 1 — Materials and Methods: Isolation and cultivation of human bone marrow stromal cells (hBMSC) Gene expression analysis. Figure S1. Osteogenic differentiation potential of hBMSC (TNAP activity) itemized to gender and age groups. Figure S2. Correlation of TNAP inducibility to frequency of certain inducibility values itemized to age groups Figure S3. Correlation of donor age with TNAP activity and inducibility itemized to gender. Figure S4. Influence of passaging on cell number and doubling time. [file 7842191.f1.pdf]

Supplemental information

Material and methods

Isolation and cultivation of human bone marrow stromal cells (hBMSC)

hBMSC were isolated from bone marrow aspirates. Briefly, about 10 ml of one marrow aspirate were diluted 1:5 in phosphate-buffered saline (PBS) (Gibco via ThemoFisher Scientific, Germany). About 20 ml of the diluted bone marrow were applied to a Percoll (Biochrom, Berlin, Germany) density gradient (d = 1.073 g/ml) and centrifuged at 900xg for 30 min at 25 °C. Mononuclear cells in the interface were harvested, filtered through a nylon cell strainer (100 µm, Becton Dickinson, Heidelberg, Germany), and washed twice with 0.5% human serum albumin in PBS. The cells were re-suspended in Dulbecco's modified essential medium (DMEM) (Gibco) containing 10% heat-inactivated fetal calf serum (HI-FCS; Biochrom, Berlin, Germany) and seeded into 75 cm² cell culture flasks (T75, Greiner bio-one, Frickenhausen, Germany) and incubated in 5% CO_2-atmosphere at 37°C. After 24 h, non-adherent cells were removed by washing with PBS. The medium was changed twice per week. These passage 0 (P0; after isolation) cells need about 3-4 weeks to become confluent.

Gene expression analysis

For analysis of gene expression real time PCR was performed according to following protocol. After an initial activation step for 5 min at 95°C, 50 PCR cycles were run (denaturation at 95°C for 5 sec; annealing and synthesis at 60°C for 10 sec). Primers were constructed by use of *Universal Probe Library* (Roche, Mannheim Germany) (see Table 1 for detailed information). Identity of the PCR products was verified by sequence analysis. The same single stranded cDNA was used to analyze the expression of the gene of interest and the house-keeping gene beta-actin. The relative expression values were counted using the

comparative quantification method of the RotorGene software release 6.0. For quantitation, values are normalized to hBMSC in P2 in BM (passaging) or hBMSC in BM at day 1 (time course).

Supplemental figure captures

Figure 1S1 ***Osteogenic differentiation potential of hBMSC (TNAP activity).*** hBMSC in passage 1 were plated in BM (basic medium) onto TCPS (=P2). From day 4 after plating, the cells were cultured either in BM or in OM/D (osteogenic differentiation medium) and analyzed at day 15 after plating for TNAP activity with p-nitrophenylphosphate as a substrate; the calculation of TNAP activity was performed with a linear calibration curve obtained with p-nitrophenolate (*r=0.9979*). TNAP activity was normalized to the protein content of the lysates. The donors were grouped according their age (and gender) into three subgroups (18-25yrs., 26-25 yrs., and > 35 yrs.) and analysed separately for statistical significance. Significant differences of OM/D mean vs. BM mean values were analysed by paired t-test and indicated with c ($p<0.001$); the number of analysed donors is indicated in the graphs.

Figure 1S2 ***Osteogenic differentiation potential of hBMSC (comparison of females and males groups).*** hBMSC in passage 1 were plated in BM (basic medium) onto TCPS (=P2). From day 4 after plating, the cells were cultured either in BM or in OM/D (osteogenic differentiation medium) and analyzed at day 15 after plating for TNAP activity as described. The donors were grouped according their age into three subgroups (18-25yrs., 26-25 yrs., and > 35 yrs.) and analysed for statistical differences of endogenous TNAP activity, TNAP activity in OM/D, and inducibility. Significant differences between females and males were analysed by unpaired t-test and indicated with ***b*** ($p<0.01$) and ***c*** ($p<0.001$); the number of analysed donors is given in table 2.

Figure 1S3 ***Osteogenic differentiation potential of hBMSC (comparison of age groups).*** hBMSC in passage 1 were plated in BM (basic medium) onto TCPS (=P2). From day 4 after plating, the cells were cultured either in BM or in OM/D (osteogenic differentiation medium) and analyzed at day 15 after plating for TNAP activity as described. The donors were grouped

according their age into three subgroups (18-25yrs., 26-25 yrs., and > 35 yrs.) and analysed for statistical differences of endogenous TNAP activity, TNAP activity in OM/D, and inducibility. Significant differences between age groups were analysed by one-way ANAOVA with Bonferroni's post-test and indicated with ***b*** (p<0.01); the number of analysed donors is given in table 2.

Figure 2S ***Correlation of TNAP inducibility to frequency of certain inducibility values.*** hBMSC in passage 1 were plated in BM (basic medium) onto TCPS (=P2). From day 4 after plating, the cells were cultured either in BM or in OM/D (osteogenic differentiation medium) and analyzed at day 15 after plating for TNAP activity as described. For the age groups the inducibility of TNAP activity (TNAP activity in OM/D:TNAP activity in BM) was plotted against the frequency of certain inducibility value The data points were analysed by non-linear curve fit and resulted in a Gaussian distribution. The peak characteristics (x_{max}, y_{max}) are indicated in the graphs; the number of donors is given in graphs.

Figure 3S ***Correlation of donor age with TNAP activity and inducibility.*** hBMSC in passage 1 were plated in BM (basic medium) onto TCPS (=P2). From day 4 after plating, the cells were cultured either in BM or in OM/D (osteogenic differentiation medium). At day 15 after plating, the cells were analyzed for TNAP activity as described above. Data for endogenous TNAP activity, TNAP activity in OM/D, and TNAP inducibility separately for females and males donor age group were plotted each in one graph. The lines indicate linear curve fit. Regression characteristics (p-value, R-square) and the number of analyzed donors are given in table 2.

Figure 4S ***Influence of passaging on cell number and doubling time.*** hBMSC in passage 1 were plated in BM (basic medium) onto TCPS (=P2), trypsinized and counted after 7 days

in culture. Then the cells were re-plated in the same density for the next passage until P10. Cell numbers (white columns) and calculated doubling time (black columns) of each passage are shown as mean ± SEM, n=4.

Supplemental figures

Figure 1S1

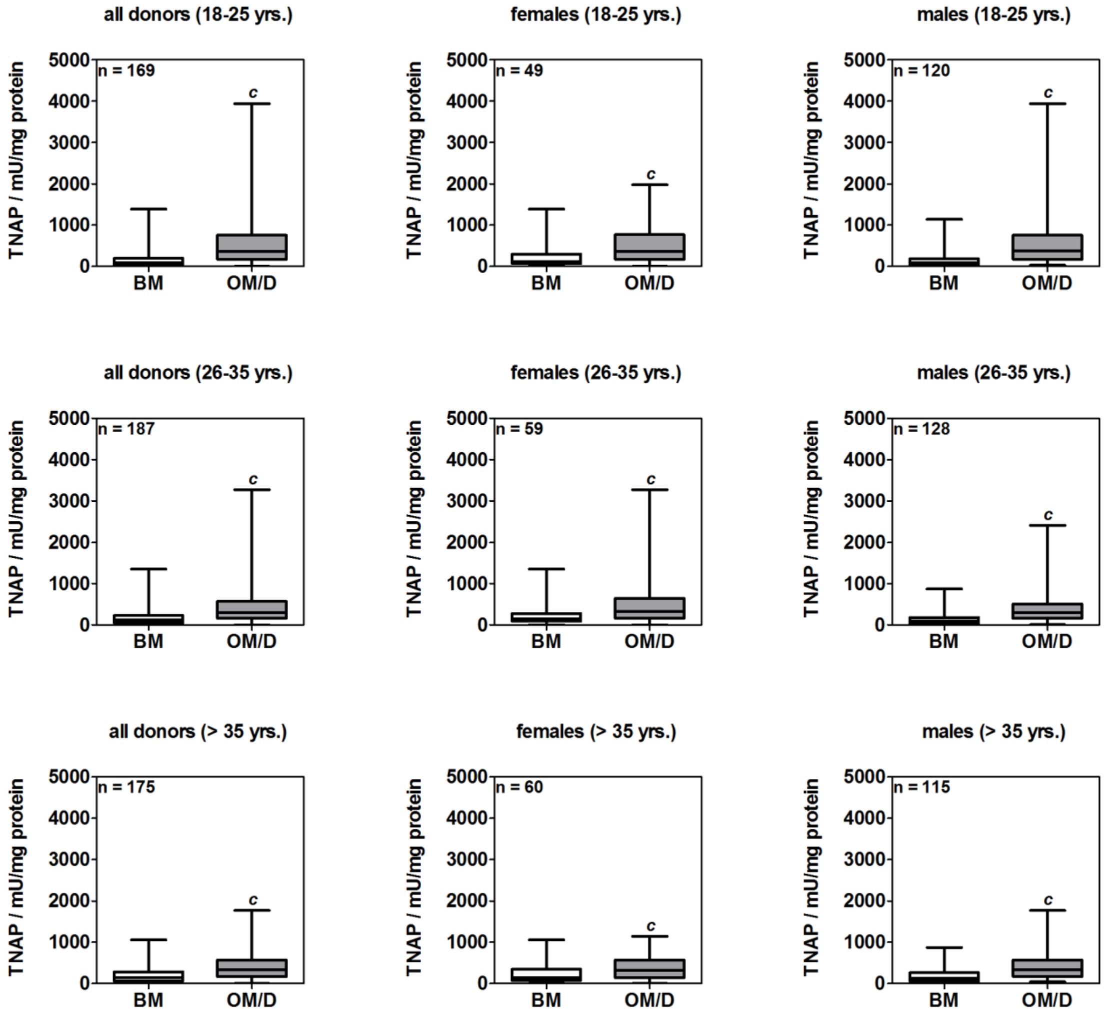

Figure 1S2

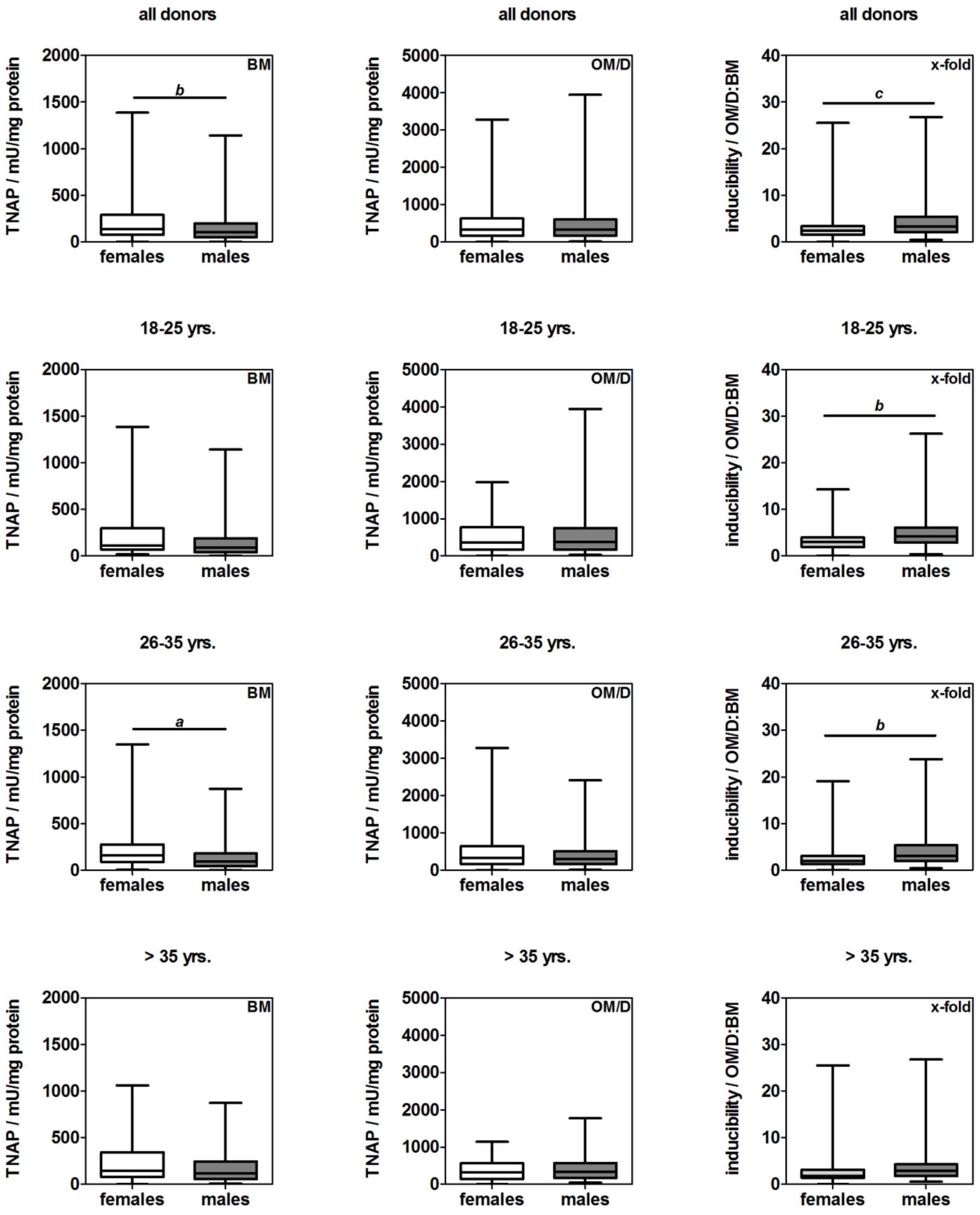
all donors
18-25 yrs.
26-35 yrs.
> 35 yrs.
TNAP / mU/mg protein
inducibility / OM/D:BM
BM
OM/D
x-fold
females
males
a
b
c
0
500
1000
1500
2000
3000
4000
5000
10
20
30
40

Figure 1S3

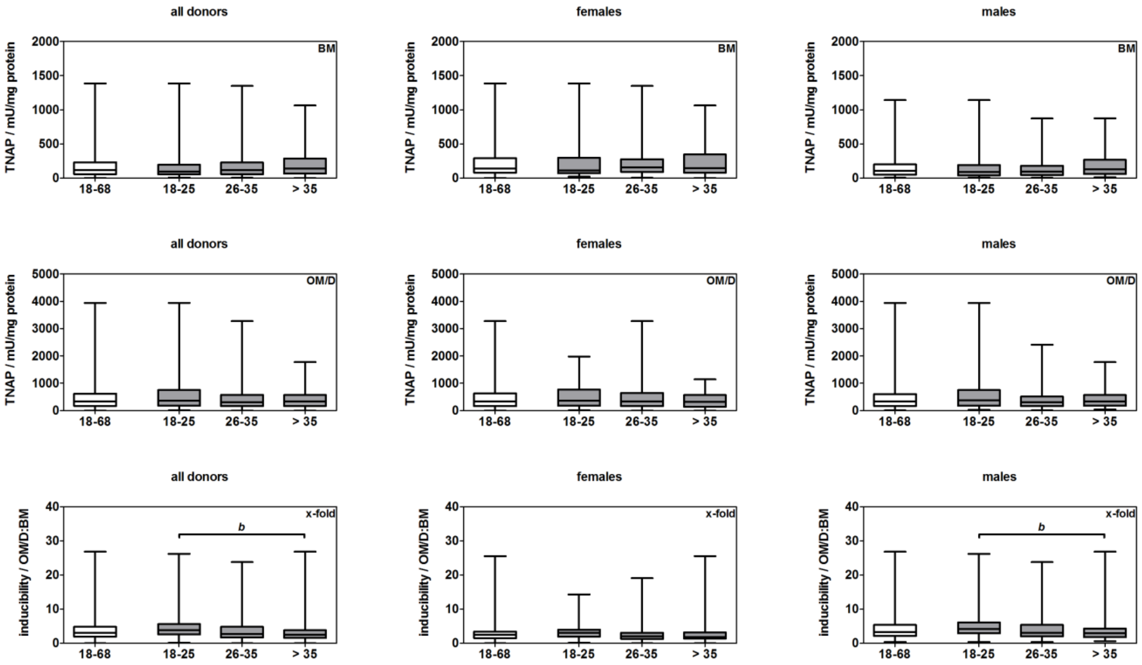

all donors
females
males
BM
TNAP / mU/mg protein
2000
1500
1000
500
0
18-68
18-25
26-35
> 35
OM/D
5000
4000
3000
2000
1000
x-fold
inducibility / OMD:BM
40
30
20
10
b

Figure 2S

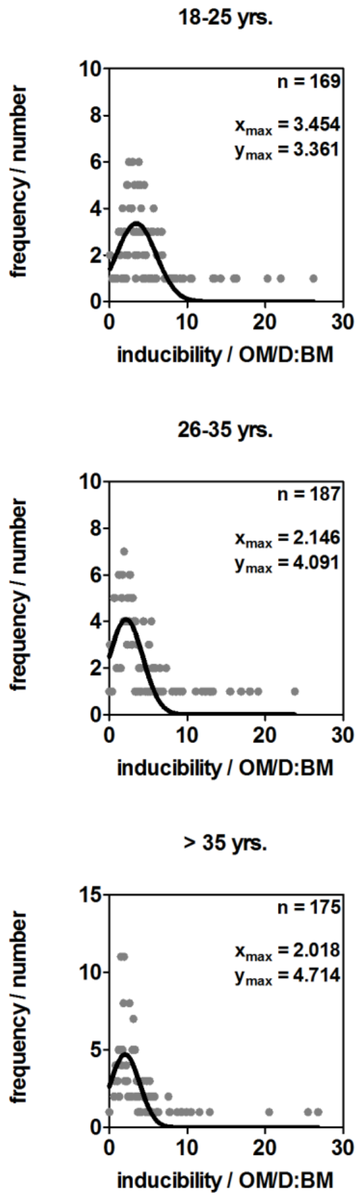
18-25 yrs.
n = 169
x_{max} = 3.454
y_{max} = 3.361
frequency / number
inducibility / OM/D:BM
26-35 yrs.
n = 187
x_{max} = 2.146
y_{max} = 4.091
frequency / number
inducibility / OM/D:BM
> 35 yrs.
n = 175
x_{max} = 2.018
y_{max} = 4.714
frequency / number
inducibility / OM/D:BM

Figure 3S

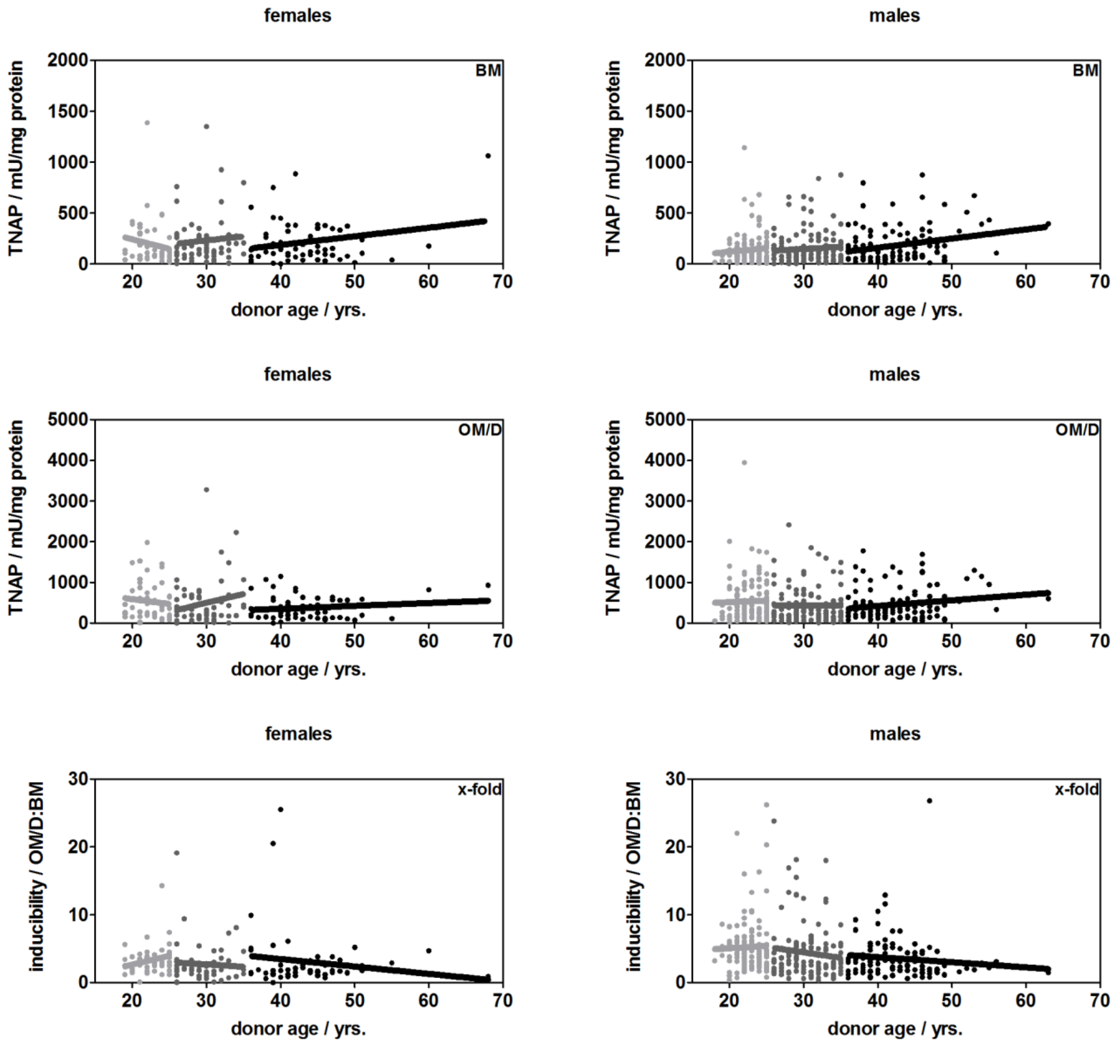
females
males
BM
OM/D
x-fold
TNAP / mU/mg protein
inducibility / OM/D:BM
donor age / yrs.
2000
1500
1000
500
0
5000
4000
3000
2000
1000
0
30
20
10
0
20
30
40
50
60
70

Figure 4S

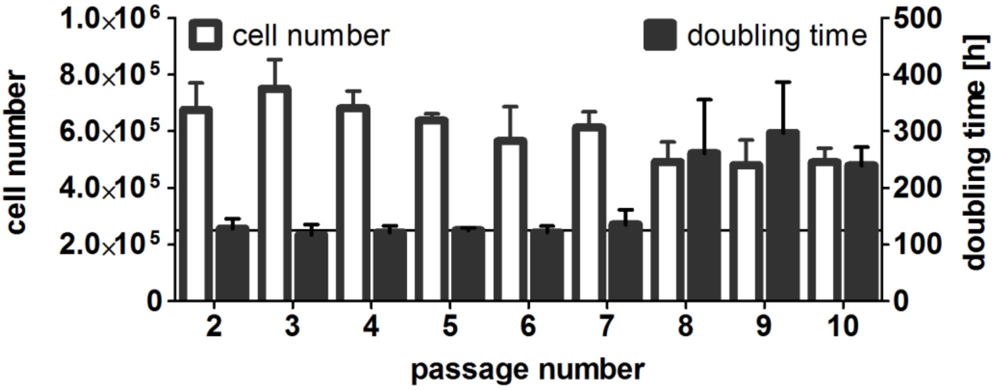

cell number
doubling time
1.0×10 6
8.0×10 5
6.0×10 5
4.0×10 5
2.0×10 5
0
500
400
300
200
100
0
cell number
doubling time [h]
2
3
4
5
6
7
8
9
10
passage number
